# Macrovascular Complications in Patients with Diabetes and Prediabetes

**DOI:** 10.1155/2017/7839101

**Published:** 2017-11-07

**Authors:** Dou Huang, Marwan Refaat, Kamel Mohammedi, Amin Jayyousi, Jassim Al Suwaidi, Charbel Abi Khalil

**Affiliations:** ^1^Department of Medicine and Genetic Medicine, Weill Cornell Medicine-Qatar, Doha, Qatar; ^2^Department of Internal Medicine, Cardiovascular Medicine/Cardiac Electrophysiology, American University of Beirut Faculty of Medicine and Medical Center, Beirut, Lebanon; ^3^Department of Diabetology, Endocrinology and Nutrition, Bichat Hospital, Assistance Publique Hôpitaux de Paris, 75877 Paris, France; ^4^Department of Diabetes, Hamad Medical Corporation, Doha, Qatar; ^5^Adult Cardiology, Heart Hospital, Hamad Medical Corporation, Doha, Qatar

## Abstract

Diabetes is a significant health problem worldwide, and its association with cardiovascular disease (CVD) was reported in several studies. Hyperglycemia and insulin resistance seen in diabetes and prediabetes lead to an increase in reactive oxygen species, which triggers intracellular molecular signaling. The resulting prothrombotic state and increase in inflammatory mediators expedite atherosclerotic changes and the development of macrovascular complications. Individuals with diabetes or prediabetes have a higher risk of developing myocardial infarction, stroke, and peripheral artery disease. However, no significant difference in cardiovascular morbidity has been observed with tight glycemic control despite a reduction in some CVD outcomes, and the risk of adverse outcomes such as hypoglycemia was increased. Recently, some GLP-1 receptor agonists and SGLT-2 inhibitors have been shown to reduce cardiovascular events and mortality. In this review we give an overview of the risk and pathogenesis of cardiovascular disease among diabetic and prediabetic patients, as well as the implication of recent changes in diabetes management.

## 1. Introduction 

Diabetes has been recognized as a global epidemic, with the number of adults with diabetes reaching 422 million and an estimated prevalence of 8.5% worldwide in 2014 [1]. However, the prevalence of diabetes is heterogeneous and varies according to nations. In Arabic countries such as Qatar, it is estimated to be 20.2%, whereas in the United States the prevalence is about 12.3% [2, 3], suggesting a more gravid healthcare burden and more pressing issue.

Diabetes is a leading cause of microvascular complications such as nephropathy and retinopathy. It is also associated with an accelerating atherosclerosis, and type 2 diabetes mellitus (T2DM) is usually not detected until late in the course of cardiovascular disease (CVD). Therefore, many patients are suffering from complications at or shortly after diagnosis. The strong association between diabetes and CVD was observed in multiple studies, independently of other traditional cardiovascular risk factors [4–7]. Being the most common cause of mortality in diabetic patients, CVD mortality accounts for 52% of deaths in T2DM and 44% in type 1 diabetes mellitus (T1DM) [8].

Recently, prediabetic states, characterized by impaired fasting glycaemia (IFG) or impaired glucose tolerance (IGT), have also been shown to be associated with CVD morbidity and mortality [9, 10]. It is therefore important to have a better understanding of the pathophysiology, in order to identify new approach to tackle or prevent the development of macrovascular complications early on. This article attempts to review current understanding of the epidemiology, pathogenesis, and implication of increased CVD risk in diabetic and prediabetic population.

## 2. Pathogenesis of CVD in Diabetes Mellitus

Hyperglycemia and insulin resistance, among various other factors, are thought to contribute significantly to atherosclerotic changes and the pathogenesis of macrovascular complications in diabetes. Though both are commonly observed in diabetic patients, insulin resistance usually develops years before hyperglycemia becomes clinically significant.

### 2.1. Insulin Resistance

Obesity plays an important part in the pathogenesis of insulin resistance, which is commonly seen in T2DM patients. By releasing free fatty acids (FFAs) and inflammatory mediators, adipose tissue alters lipid metabolism, increases reactive oxygen species (ROS) production, and increases systemic inflammation [11]. Insulin resistance is related to abnormal function of the glucose transporter type 4 (GLUT-4), the insulin-mediated glucose transporter mainly found in adipose cells and muscle cells. When FFAs bind to Toll-like receptor (TLR), PI3-kinase (PI3K) and Akt activity are downregulated, which reduces expression of GLUT-4 [12], leading to decreased response to insulin binding.

**Figure 1:**
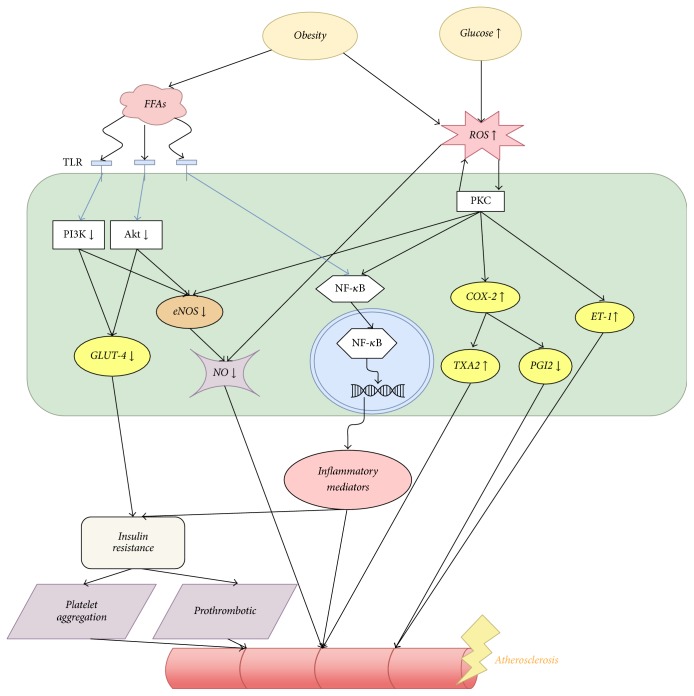
*The effect of insulin resistance and hyperglycemia in CVD pathogenesis*. Insulin resistance is tightly correlated with obesity, which increases FFA and ROS level, both of which contribute to atherosclerotic changes and the development of macrovascular complications. Increased plasma glucose level contributes to increased production of ROS as well, which activates PKCs intracellularly and leads to inflammatory changes and atherosclerosis. FFAs: free fatty acids, ROS: reactive oxygen species, TLR: Toll-like receptor, PI3K: PI3-kinase, PKC: protein kinase C, eNOS: endothelial nitric oxide synthase, NO: nitric oxide, COX-2: cyclooxygenase-2, TXA2: thromboxane A2, PGI_2_: prostacyclin, ET-1: endothelin-1.

Meanwhile, decreased PI3K and Akt activity also lead to inactivation of endothelial nitric oxide synthase (eNOS), which reduces nitric oxide (NO) production [13]. NO activity is further reduced by increased ROS generation caused directly by obesity and insulin resistance, due to the NO-inactivating effect of ROS. NO is a key molecule in maintaining normal function of endothelial cells. Obesity and insulin resistance induced decrease in NO activity, thus contributing to endothelial dysfunction and subsequent atherosclerotic changes (Figure 1).

In addition to downregulation of PI3-kinase and Akt, the binding of FFAs to TLR also activates nuclear factor NF-*κ*B, which triggers transcription of inflammatory molecules, contributing to insulin resistance and atherosclerosis development [12]. The blockade of NF-*κ*B in a mice model resulted in decrease in systemic oxidative markers, adhesion molecule gene expression, and macrophage infiltration, processes that contribute to atherosclerosis [14], suggesting an important upregulation role of NF-*κ*B in CVD development.

Parallel to atherosclerotic changes, thrombosis also plays an important role in the development of macrovascular complications in diabetes. In physiological setting, insulin inhibits thrombosis and increases fibrinolysis, and insulin resistance creates a prothrombotic state [15]. Lack of insulin also results in calcium accumulation in platelets, which enhances platelets aggregation [16], further contributing to CVD development.

### 2.2. Hyperglycemia

Hyperglycemia is also involved in the pathogenesis of cardiovascular complication of diabetes. It increases the production of ROS, which inactivates NO [17], leading subsequently to endothelial dysfunction. On the other hand, increased ROS production contributes to CVD by triggering the activation of protein kinase C (PKC). Acting as a group of enzymes that can affect the function of other cellular proteins, PKC has been shown to have an effect on vascular cell growth and apoptosis, permeability, extracellular matrix synthesis, and cytokine production [18]. Activation of PKC results in alteration of vascular homeostasis and predisposition to vascular complications. PKC in turn induces ROS production in vascular cells [19], perpetuating the vicious cycle (Figure 1).

PKC also affects endothelial cells in different molecular aspects, including inactivation of NO and overproduction of vasoconstrictors. As mentioned above, PKC increases production of ROS, which decreases NO availability. At the same time, PKC directly decreases eNOS activity, by inhibiting eNOS gene expression [20]. PKC also induces vasoconstrictor synthesis: the production of endothelin-1 (ET-1), a molecule involved in platelet aggregation and vasoconstriction, is upregulated by PKC activation [18]; PKC enhances activity of cyclooxygenase-2 (COX-2) expression, which increases thromboxane A2 (TXA2) and decreases prostacyclin (PGI_2_) production. The combination of reduced NO availability and increased vasoconstrictor production promotes the development of vascular atherosclerotic changes.

Hyperglycemia and PKC activation-induced ROS production causes inflammatory changes in vascular endothelium. With increased ROS level, the nuclear factor NF-*κ*B subunit p65 expression and nuclear translocation are upregulated, leading to increased transcription of genes encoding inflammatory factors [21]. The increased production of inflammatory mediators leads to monocytes adhesion, extravasation, and formation of foam cells, further contributing to the development of atherosclerosis. Chronic hyperglycemia is also responsible for cardiovascular damage through activation of other major biochemical paths including polyol pathway flux, increased formation of advanced glycation end products (AGEs), increased expression of AGEs receptor and its activating ligands, and overactivity of the hexosamine pathway [22].

## 3. Risk of Coronary Heart Disease among Patients with Diabetes

Diabetes is associated with increased risk of coronary heart disease (CHD). In patients without prior history of myocardial infarction (MI), the 7-year risk of MI is 20.2% and 3.5% for diabetics versus nondiabetics, respectively. Similarly, in patients with a history of MI, the 7-year risk of MI is 45.0% and 18.8% for diabetics and nondiabetics, respectively [22]. The 7-year risk of developing MI in diabetic patients was comparable to the risk of MI in nondiabetic patients who have had a prior MI, which suggests that diabetes contributes significantly to the development of MI and can possibly be considered as a CHD risk equivalent. However, a population study, which included adult residents in Denmark who are 30 years or older, showed that diabetes increased the risk of CHD but not to the extent of a risk equivalent during the 5-year follow-up. In this study, men with diabetes had a hazard ratio (HR) of 2.30 for developing MI, which was lower than the risk of nondiabetic men with a history of prior MI (in whom HR = 3.97). Similar findings were observed for CHD mortality, incidence of total CVD events, and cardiovascular mortality [23]. In a meta-analysis consisting of 13 studies, diabetic patients who did not have a history of MI have a 43% lower risk of developing CHD compared to nondiabetics with prior history of MI [24].

Diabetes has also a negative impact on the treatment of CHD. When evaluating percutaneous coronary intervention performed in patients with ST-elevation MI, those with diabetes had a higher 3-year risk of target lesion revascularization, MI recurrence, and all-cause mortality, as compared to those without diabetes [25]. The analysis of patients treated with drug-eluting stent after MI showed that diabetes is more prevalent in patients who developed stent thrombosis than in those who did not [26]. Diabetic patients are 1.8 times more likely to develop stent thrombosis than nondiabetic patients 1 year after stenting [27]. In terms of coronary artery bypass graft (CABG) surgery, patients with diabetes had a significantly higher operative mortality, with a relative risk of 1.67 compared to nondiabetics [28]. Interestingly, by achieving intensive antiplatelet effect with prasugrel, patients with diabetes have a more significant reduction of MI incidence compared to those without diabetes [29]. This finding may suggest a significant role of platelet activation and aggregation in the development of CHD in diabetes.

## 4. Risk of Stroke among Diabetics

Other than CHD, diabetes also increases the risk of stroke. The INTERSTROKE study, which is a case-control study that recruited patients who developed acute stroke and those without a stroke history in 22 countries, demonstrated a 35% increase in stroke risk in patients with self-reported history of diabetes [30]. In a meta-analysis with 102 prospective studies, diabetic patients had a 2.3-time higher risk of developing ischemic stroke and a 1.6-time higher risk of developing hemorrhagic stroke compared to nondiabetics [31].

Diabetes is also correlated with a worse outcome and more disability after stroke. Among patients admitted for acute stroke, diabetes was associated with a higher risk of death or functional dependency (characterized by modified Rankin Scale of 3–6) [32]. The Australian Stroke Unit Registry demonstrated a worse functional outcome 3 months after acute stroke in patients with diabetes compared to those without [33]. Patients with impaired fasting glycaemia also showed a poor functional outcome and a lower rate of discharge to home after acute stroke [34].

Stroke recurrence is also affected by diabetes. The Dutch TIA trial, which studied patients who developed minor ischemic stroke or transient ischemic attacks (TIAs), showed a 2.10-time higher risk of developing nonfatal stroke among diabetic patients compared to nondiabetic subjects [35].

## 5. Risk of Peripheral Arterial Disease among Diabetics

Peripheral arterial disease (PAD) is a common macrovascular complication in patients with diabetes. The German Epidemiological Trial on Ankle Brachial Index (GETABI) study demonstrated that among patients aged 65 or older, diabetic patients have a 2-fold higher rate of PAD (defined as ABI < 0.9), as well as a 2.5-fold higher risk of intermittent claudication [36]. In patients diagnosed with PAD, the risk of developing an ischemic ulceration is increased by more than 20% in 10 years, with a 3-fold higher likelihood among diabetics. Moreover, 30% of the patients were found to have ischemic rest pain during the follow-up, and diabetes increased the risk by 1.8-fold [37].

ABI is useful in identifying PAD and quantifying PAD severity. The value 0.9 has been used as a cut-off for signs of arterial occlusion. However, diabetic patients may have certain degrees of arterial occlusion at a higher ABI value, which results in underdiagnosis of PAD in this population [38]. A study demonstrated that the cut-off value with the highest sensitivity and specificity for diabetic patients is somewhere between 1.0 and 1.1 [39]. The sensitivity of ABI is significantly limited in diabetic patients compared to nondiabetics, which could be partially explained by the arterial stiffness resulting from medial artery calcification [40]. Calcification causes the vessels to be poorly compressible and increases ABI. In fact, in diabetic patients, particularly those with impaired renal function, ABI > 1.4 is also suggestive of PAD [41].

Diabetes is also associated with worse revascularization outcomes [42]. In addition to the higher risk of limb loss, there is also a significant increase in cardiovascular event rates in patients with PAD. Decreased ABI has been shown to be an independent risk factor for CVD event, cardiovascular mortality, and overall mortality. In a retrospective follow-up of over 450 patients with T2DM, ABI less than 0.9 was associated with a significant increase in the primary composite endpoint of major cardiovascular events and in the secondary endpoint of all-cause mortality, compared to ABI equal to or higher than 0.9 [43]. In a similar prospective cohort of 3000 Japanese individuals, a low ABI was independently associated with a higher incidence of cardiovascular events and mortality in patients with and without T2DM [44].

## 6. Relation of CVD with Prediabetes

Disturbed glucose metabolism plays a major role in atherosclerosis and CVD. Cumulative data are suggesting that increased plasma glucose level is a risk factor for CVD regardless of the presence of diabetes. A prediabetic state could be defined by IFG (fasting glucose level of 5.6–6.9 mmol/l), IGT (2-hour postcharge glucose of 7.8–11.0 mmol/l), and/or HbA_1c_ level of 5.7%–6.4% [45].

Compared to those with a fasting glucose level of 3.90–5.59 mmol/l, those with a level higher than 5.60 mmol/l (i.e., prediabetic or diabetic) have an increased risk of developing CHD [31]. In the Heart Outcomes Prevention Evaluation (HOPE) study, the risk of cardiovascular events (MI, stroke, and cardiovascular death) in the following 4.5 years increases by almost 9% with every 1 mmol/l increase in fasting glucose. Every 1% increase in HbA_1c_ was also correlated with a higher risk of cardiovascular outcomes, with a relative risk of 1.07. These relationships were independent of other cardiovascular risk factors (age, sex, blood pressure, and hyperlipidemia) and remained significant after adjustment for diabetic status [9]. Similarly, the Diabetes Epidemiology: Collaborative analysis Of Diagnostic criteria in Europe (DECODE) study showed a correlation between fasting plasma glucose and CVD-associated mortality, independently of diabetic status. The relationship between fasting plasma glucose and CVD-associated mortality seemed to be “J-shaped” curve, with no threshold effect observed at high glucose level [46].

However, the relation between CVD and fasting glucose level in the prediabetic range is not consistent among studies. The Hoorn study, a cohort study in the Dutch population, demonstrated that fasting glucose levels are correlated with cardiovascular mortality in the diabetic range, but not in the prediabetic range. However, the same study showed that postprandial glucose level and HbA_1c_ levels predict an increase in the 8-year risk of cardiovascular mortality, in both diabetics and nondiabetics [47].

IGT and HbA_1c_ appear to correlate more with CVD risk than IFG. The Funagata Diabetes Study, a cohort study in Japanese population, observed a correlation between CVD and IGT, but not with IFG [48]. The Framingham Offspring study made similar observations [49]. When analyzed separately, CVD incidence during the 4-year follow-up correlated with fasting glucose, glucose tolerance, and HbA1c, with a relative risk of 1.13 for every 0.7 mmol/l increase in fasting glucose, 1.26 for every 2.1 mmol/l increase in postprandial glucose, and 1.24 for every 0.7% increase in HbA1c. When analyzed in the same model, fasting glucose had a much weaker effect, while postprandial glucose still significantly increases CVD risk. In a meta-analysis comprised of 53 cohort studies, patients with prediabetic states were found to be at an increased risk for CVD, CHD, and stroke. Patients with IGT had a higher risk compared to those with IFG [50]. In a prospective study of nondiabetic patients admitted for MI with a blood glucose level < 11.1 mmol/l, 35% of the patients were found to have IGT at discharge. At 3-month follow-up, 31% fulfilled the criteria of diabetes [51]. Another study with a larger patient population reached similar conclusions. Among the patients admitted for an acute coronary syndrome, 36% were found to have IGT and 22% previously undiagnosed diabetes [52].

## 7. Recent Trend

During the last decade, with better recognition of the adverse effect imposed by diabetes and availability of novel pharmacological reagents, we have observed better control of glycemia, HbA_1c_, blood pressure, and lipid profile in diabetic patients. Meanwhile, the risk of CVD has significantly decreased. With the UK Prospective Diabetes Study (UKPDS) algorithms, the estimated 10-year risk for CHD among diabetic patients was 21.1% in the period of 1999-2000, which has decreased to 16.4% in 2007-2008 [53]. In the US adult population, the CVD-associated mortality rate among diabetic patients decreased by 40% from 1997-1998 to 2003-2004, while the diabetes-associated excess CVD-associated mortality rate was reduced by 60% [54]. A similar trend was observed with the Swedish population, where modifiable CHD risk among diabetics decreased from 37.7% in 2003 to 19.1% in 2008 [55].

The prevalence and outcome of stroke have also improved over the past few decades. Between 1992 and 2002, the incidents of first CVD—including CHD and ischemic stroke—decreased in patients with diabetes in Finland [56]. Another study showed that although mortality after the first ischemic stroke is higher in patients with diabetes than nondiabetics, the mortality rate among diabetes has declined over the study period of 1988–2002 [57].

Unlike CHD and stroke, the prevalence of PAD among diabetics was not significantly different with intensive treatment of diabetes in addition to current standard diabetic care [58]. The incidence of PAD varied among studies. A study in Queensland demonstrated that between 2005 and 2010, the incidence of hospitalization related to PAD among diabetics has decreased by 43%, and the incidence of amputation has decreased by 40% [59]. However, a Spanish study showed a significant increase in lower limb amputation rate in patients with T2DM from 2001 to 2008 [60]. The discrepancy could be due to different quality of care for diabetes-related foot conditions, among other factors. Data from 84 hospitals in Los Angeles showed significant variability in rates of lower extremity amputation between different types of hospitals [61].

## 8. The Effect of Glycemic Control on Macrovascular Complications 

The association of diabetes and prediabetes with CVD and recent changes in CVD prevalence among diabetes suggests the possibility of preventing CVD development by better controlling diabetes. Considering the role of hyperglycemia in CVD pathogenesis, tight glycemic control seems to be a reasonable approach for many decades, which has been investigated by multiple clinical trials (Table 1).

The Action to Control Cardiovascular Risk in Diabetes (ACCORD) study randomized diabetic patients to an intensive therapy group with targeting HbA_1c_ < 6.0% and a group receiving standard therapy with targeting HbA_1c_ 7.0–7.9%. In 1 year, the patients with intensive glycemic control had a reduced incidence of CVD, but not statistically significant reduction of macrovascular events. Meanwhile, mortality, incidence of hypoglycemic events, and weight gain were significantly higher in the group receiving intensive glycemic control compared to that receiving standard therapy [62]. Similarly, the Action in Diabetes and Vascular Disease: Preterax and Diamicron MR Controlled Evaluation (ADVANCE) trial compared standard treatment with intensive glycemic control, which involved the use of gliclazide and other hypoglycemic agents as needed. 11,140 patients who have been diagnosed with T2DM were randomly assigned to two groups. The intensive treatment group achieved an average HbA_1c_ of 6.5% compared to 7.3% in the control group in median 5-year follow-up. The study showed no significant difference in the incidence of macrovascular complications, cardiovascular mortality, and overall mortality between the two groups. However, the incidence of severe hypoglycemia was higher in the intensive glycemic control group, with a hazard ratio of 1.86 [63]. Comparable outcomes were observed in the Veterans Affairs Diabetes Trial (VADT). The trial recruited 1791 military veterans, whose T2DM was not optimally controlled. The patients were randomized to standard treatment group and intensive therapy group. At a median 5.6-year follow-up, there was no significant difference in major CVD, CVD-associated mortality, and other macrovascular complications. The rate of adverse events, mainly hypoglycemia, was significantly higher in the intensive therapy group compared to the standard treatment group [64].

Furthermore, two meta-analyses demonstrated that intensive glucose control reduced the incidence of cardiovascular events, particularly nonfatal MI. The CVD-associated mortality and overall mortality, however, were not significantly different, and the risk of hypoglycemia is higher in the intensive therapy group than the standard treatment group [65, 66]. The observations from the above-mentioned trials lead to the conclusion that intensive glycemic control alone is not enough to prevent macrovascular complications. Nevertheless, an approach based on multiple risk control of macrovascular disease offers a reduction in mortality and macro- and microvascular events as demonstrated by the STENO-2 study [67, 68].

Insulin resistance also plays a role in CVD pathogenesis and is therefore a possible therapeutic target. In diabetic patients with established atherothrombosis, metformin treatment reduced the all-cause mortality rate from 9.8% to 6.3% [69]. In the UKPDS study, patients who had been recently diagnosed with diabetes were randomized to a dietary restriction group or an intensive treatment group. The intensive treatment group involves the use of sulfonylurea, insulin, or metformin. Patients on metformin had less reduction of HbA_1c_ levels compared to those on sulfonylurea or insulin. However, a greater reduction of MI and overall mortality was observed in the metformin group compared to the sulfonylurea group at 5-year follow-up [70].

However, this neutral trend was recently reversed with new antidiabetic medications such as the GLP-1 receptor agonists and the SGLT-2 inhibitors. It is worth noting that those studies were not designed to test whether an intensive glycemic treatment would reduce cardiovascular events compared to a conventional one, but rather to assess cardiovascular safety. In the Liraglutide Effect and Action in Diabetes: Evaluation of Cardiovascular Outcome Results (LEADER) trial, liraglutide was superior to placebo in reducing major cardiovascular events when added to the conventional treatment of individuals with T2DM at high cardiovascular risk [71]. A similar decrease in major events was also reported in the Semaglutide and Cardiovascular Outcomes in Patients with Type 2 Diabetes (SUSTAIN-6) trial despite a relatively short follow-up period (mean: 2.1 years) and an HbA_1c_ > 7.5% at the end of the trial in both groups [72]. However, 2 other GLP-1 agonist trials did not show a reduction in cardiovascular events: the Evaluation of Lixisenatide in Acute Coronary Syndrome* (ELIXA)* [73], which randomized T2DM patients post-MI to lixisenatide or placebo, and the recently published Exenatide Study of Cardiovascular Event Lowering (EXSCEL) trial that failed to demonstrate a cardiovascular benefit of weekly injections of exenatide [74]. The Empagliflozin, Cardiovascular Outcomes, and Mortality in Type 2 Diabetes (EMPA-REG) trial is the first to assess cardiovascular outcomes of an SGLT-2 inhibitor in T2DM patients. Interestingly, empagliflozin decreased cardiovascular mortality and was also associated with a 35% risk reduction of hospitalization for heart failure [75]. In the recently published Canagliflozin and Cardiovascular and Renal Events in Type 2 Diabetes (CANVAS) trial, patients with T2DM and high cardiovascular risk experienced less cardiovascular events during the mean of 3.6 years [76]. Other SGLT-2 inhibitors and GLP-1 receptor agonists' trials are expected to be presented within the coming years.

## 9. Conclusion

In summary, both diabetes and prediabetes predispose patients to the development of macrovascular complications of diabetes, through complex molecular pathways that involve hyperglycemia and insulin resistance. While intensive glycemic control alone might not reduce mortality and major cardiovascular events, a global approach consisting of life-style modifications, decreasing hyperglycemia, and treating cardiovascular risk factors associated with diabetes is beneficial to the cardiovascular risk profile of those patients; hence, the target of blood glucose control should be tailored to the individual patients. In recent years, a new hope has risen with the new class of antidiabetic agents such as SGLT-2 inhibitors and GLP-1 receptor agonists to decrease mortality in patients with T2DM without increasing the risk of hypoglycemia.

## Figures and Tables

**Table 1:** *Intensive glycemic control and cardiovascular events in type II diabetes*. There was comparable CVD risk in the intensive glycemic control group and the standard therapy group. However, the risk of hypoglycemia is significantly higher in the groups with intensive control therapy.

Clinical trial	ACCORD		ADVANCE		VADT	
Sample size	10,251		11,140		1,791	
Median follow-up	3.5 years		5 years		5.6 years	

Treatment group	Intensive control	Standard therapy	Intensive control	Standard therapy	Intensive control	Standard therapy

Mean HbA1c	6.7%	7.5%	6.5%	7.3%	6.9%	8.4%
CVD event	6.9%	7.2%	10.0%	10.6%	30.0%	34.0%
	(*p* = 0.16)		(*p* = 0.32)		(*p* = 0.14)	
CVD mortality	2.6%	1.8%	4.5%	5.2%	4.5%	3.7%
	(*p* = 0.02)		(*p* = 0.12)		(*p* = 0.29)	
All-cause mortality	5.0%	4.0%	8.9%	9.6%	11.4%	10.6%
	(*p* = 0.04)		(*p* = 0.28)		(*p* = 0.62)	
Hypoglycemia	10.5%	3.5%	2.7%	1.5%	1333 episodes	383 episodes
	(*p* < 0.001)		(*p* < 0.001)		(*p* < 0.001)	
